# Expression and Role of INSL3 in the Fetal Testis

**DOI:** 10.3389/fendo.2022.868313

**Published:** 2022-04-06

**Authors:** Richard Ivell, Linn Salto Mamsen, Claus Yding Andersen, Ravinder Anand-Ivell

**Affiliations:** ^1^ School of Bioscience, University of Nottingham, Sutton Bonington, United Kingdom; ^2^ Laboratory of Reproductive Biology, Section 5712, Juliane Marie Centre for Women, Children and Reproduction, Rigshospitalet, University Hospital of Copenhagen, Faculty of Health and Medical Sciences, University of Copenhagen, Copenhagen, Denmark

**Keywords:** leydig cell, testis descent, cryptorchidism, endocrine disruption, RXFP2

## Abstract

Insulin-like peptide 3 (INSL3) is a small peptide hormone of the insulin-relaxin family which is produced and secreted by the fetal Leydig cells in the testes only. It appears to be undetectable in female fetuses. In the human fetus INSL3 synthesis begins immediately following gonadal sex determination at weeks 7 to 8 post coitum and the peptide can be detected in amniotic fluid 1 to 2 weeks later. INSL3 acts through a unique G-protein-coupled receptor, called RelaXin-like Family Peptide receptor 2 (RXFP2), which is expressed by the mesenchymal cells of the gubernacular ligament linking the testes to the inguinal wall. The role of INSL3 in the male fetus is to cause a thickening of the gubernaculum which then retains the testes in the inguinal region, while the remainder of the abdominal organs grow away in an antero-dorsal direction. This represents the first phase of testis descent and is followed later in pregnancy by the second inguino-scrotal phase whereby the testes pass into the scrotum through the inguinal canal. INSL3 acts as a significant biomarker for Leydig cell differentiation in the fetus and may be reduced by maternal exposure to endocrine disrupting chemicals, such as xenoestrogens or phthalates, leading to cryptorchidism. INSL3 may have other roles within the fetus, but as a Leydig cell biomarker its reduction acts also as a surrogate for anti-androgen action.

## Introduction

**Figure 1:**
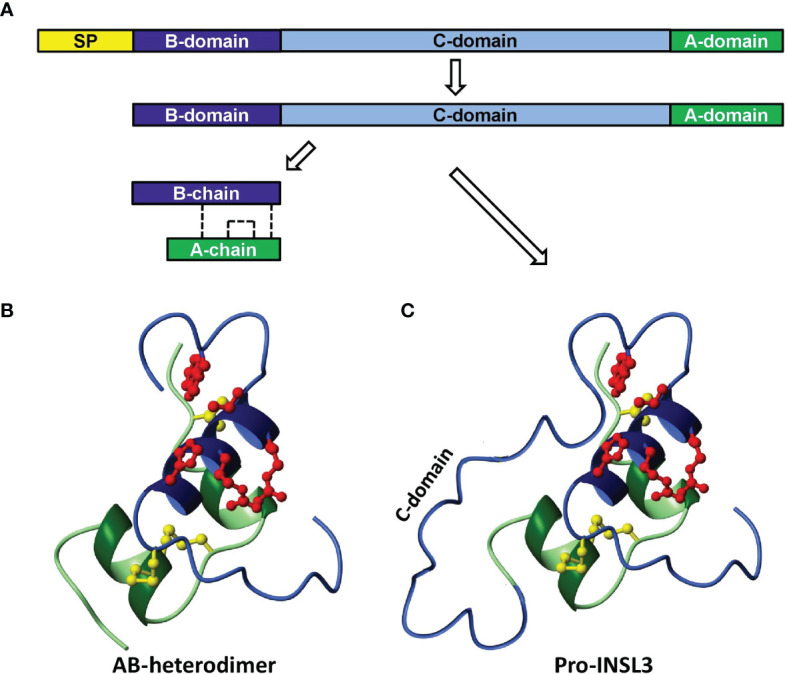
Scheme to illustrate the synthesis and processing of INSL3 from its preliminary precursor form **(A**, prepro-INSL3**)** *via* a possibly secreted pro-form **(A, C**, pro-INSL3**)**, to give rise to the final A-B heterodimer **(B)**. SP, signal peptide. The sulfhydryl bridges formed by cysteine residues are shown as dashed lines **(A)** or as yellow molecular structures **(B, C)** [reproduced from (5)].

Insulin-like peptide 3 (INSL3) was first identified as a Leydig cell-specific gene transcript encoding a putative secretory product from adult testes of boars and mice independently (1, 2) and later confirmed for other species, including human (3, 4). As its name suggests, INSL3 is a small peptide of approximately 6000 Dalton with the insulin-typical A-B heterodimeric structure (**Figure 1**), held together by three internal cysteine bonds. Its expression by the adult testes was subsequently confirmed using immunohistochemistry and immunoassay (6–10). However, it was the development of genetically altered mice lacking INSL3 expression (11, 12), which first suggested a major role for the peptide in the fetus in relation to testicular descent. Furthermore, it was the discovery of an identical phenotype of bilateral cryptorchidism in a natural mutant mouse (*Great*) which identified the receptor (RXFP2; RelaXin-like Family Peptide receptor 2) for INSL3 (13–15). Subsequent research confirmed not only the production of INSL3 by the fetal Leydig cells in rodents and the expression of RXFP2 on mesenchymal cells of the gubernacular ligament linking the fetal testes to the inguinal wall (16, 17), but also the unique action of INSL3 to achieve gubernacular thickening (7, 16, 18). Moreover, INSL3 and RXFP2 represent a unique cognate ligand-receptor pair: no other ligand at physiological concentration can activate RXFP2, and no other receptor was able to respond to INSL3 at physiological concentration (14). In retrospect, INSL3 appears to be the molecular identity of the previously mooted hormone ‘descendin’ which had been partially characterized biochemically as a factor responsible for testicular descent in fetal pigs (19).

INSL3 has now been characterized at the genome, transcript and/or the protein level from the testes of most extant mammal species, including humans. The exceptions are those mammals, such as the Afrotherian tenrec or the manatee, which appear to be primarily testicond, i.e. do not exhibit the descent of testes into a scrotum (20). INSL3 belongs to a small group of peptide hormones which have been referred to as ‘neohormones’ (21). These are hormones which, while often having ancestry in early vertebrates, have specifically evolved further in mammals to manage the specialist requirements of viviparity and internal fertilization, which required adaptations in both male and female physiology. Neohormones include several members of the relaxin-like family, including INSL3, as well as peptides like oxytocin, and proteins involved in the maternal recognition of pregnancy, such as hCG. Testicular descent became essential to provide a mechanism by which sperm could be stored in the scrotal epididymis at a temperature several degrees below abdominal core temperature. Upon ejaculation into the female tract there is a jump in temperature and environment sufficient to trigger capacitation, hyperactivation, and ultimately apoptosis unless internal fertilization occurs within the oviduct (20).

INSL3 may have roles beyond fetal life, in both adult males and females. These may be relicts of ancestral functions prior to the emergence of mammals and the acquisition of new roles and include support of gametogenesis (8, 22, 23) and bone metabolism (24), and possibly also improvement of kidney function (25). In female mammals INSL3 is expressed by the equivalent cells in the ovary to Leydig cells, the theca interna cells of growing antral follicles. Here, INSL3 is essential for the paracrine regulation of the steroid precursor androstenedione (26). These roles will not be discussed here, where the focus will be primarily on the role of INSL3 in the first phase of testis descent and cryptorchidism in the male fetus.

## The Structure of INSL3 and Its Receptor

INSL3 is encoded by a single small gene, in the human located on the short arm of chromosome 19 (19p13.11). There is a single intron separating the protein-coding domain. At its 5’ end the INSL3 gene is very close to the 3’ end of the JAK3 gene and in some species (e.g. the mouse) it even lies within a terminal intron of that gene (27). Such information suggests that all relevant regulatory sequences are likely to be restricted to only a few kilobases of genomic DNA close to the INSL3 gene. Like the genes for the structurally related peptide hormones insulin and relaxin, the INSL3 gene encodes a precursor polypeptide comprising a signal peptide to aid secretion, followed successively by an A-domain, a connecting C-domain, and finally a B-domain (**Figure 1A**). In the adult, the precursor A-C-B form is mostly processed to yield in the circulation an A-B heterodimer (**Figure 1B**), as for insulin and relaxin, although the precursor A-C-B form can also be secreted (**Figure 1C**). Importantly, both forms of INSL3 are equally bioactive at the receptor, and probably are equally detected by the immunoassays currently in use (10). For INSL3 in the fetus, we have as yet no information regarding INSL3 precursor processing and secretion for any species.

The gene for the INSL3 receptor, RXFP2, is located on the long arm of chromosome 13 (13q13.1) in the human and comprises in its full-length form 18 exons, of which the first 14 encode a long extracellular region of the G-protein-coupled receptor (GPCR), whereas the remaining 4 encode the 7-transmembrane and intracellular regions (15). Importantly, the long extracellular region includes 10 leucine-rich repeat (LRR) elements, as well as an N-terminal low density lipoprotein type a (LDLa) domain. The LRR elements are each encoded by a short 75 bp exon and are involved in the primary recognition of the INSL3 hormone *via* its B-peptide region. The LDLa domain is essential for receptor signaling and it is believed that binding of INSL3 to the receptor causes the LDLa domain to move and interact with sequences in the transmembrane region essential for signal transduction. At the same time, further parts of the INSL3 molecule, particularly in the A-peptide domain, now also appear to interact with extracellular loops of the transmembrane region of the receptor to confirm the actively signaling conformation (28).

Both INSL3 and its receptor RXFP2 are highly homologous between mammalian species, supporting their essential role in testicular descent and reproduction. Both genes, however, at least in the adult, are subject to alternative splicing (9, 29–31). The splice products, which have been identified represent mostly non-functional transcripts; for example, in the rat a rare alternative INSL3 transcript encodes an extended B-peptide only (29, 32). For RXFP2, several variant transcripts have been described; most represent forms lacking one or more of the LRR-encoding exons (31) and which consequently cannot generate a functional receptor. However, since *in vitro* evidence suggests the possibility of hetero- and homo-dimerization of this GPCR (33), it is possible that such splice variants may still be able to modulate normal receptor function.

It is important to add that, as for INSL3, we have no information at all for any species on the structure and expression of RXFP2 transcripts in the fetus. Moreover, the mRNA evidence from the fetus, mostly derived by RT-PCR, has consistently made use of only single PCR primer pairs; the application of a multiplexed PCR matrix as has been used to detect receptor splice variants in adult cells and tissues is absent.

## The Dynamics of INSL3 Expression in the Fetal Testis

**Figure 2:**
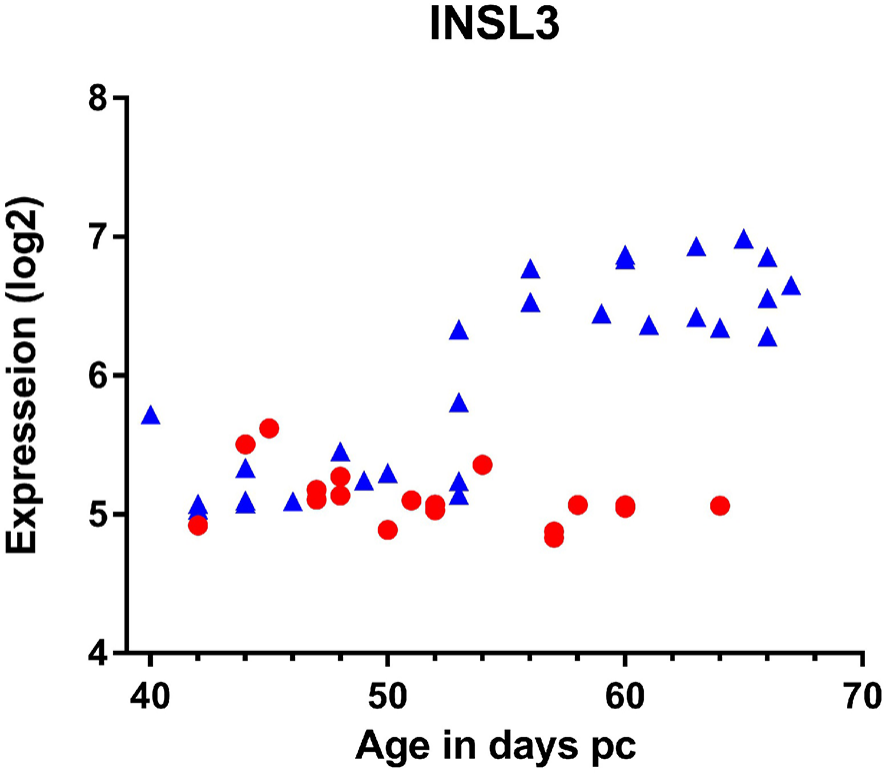
Expression of INSL3 mRNA measured by microarray analysis of gonadal tissue derived from individual male (blue triangles) or female (red circles) human fetuses at the ages indicated (35).

At least in the adult testis, INSL3 is a constitutive product uniquely of relatively mature, well-differentiated Leydig cells (32). Assuming the same is true for fetal Leydig cells, then INSL3 should be produced as soon as the fetal Leydig cells have acquired their characteristic, presumably steroidogenic phenotype. This occurs shortly after gonadal sex determination and the expression of the SRY and SOX9 genes by the fetal Sertoli cells. In humans, this would be around weeks 7-8 post coitum (pc; equivalent to gestational age (time since last menses) less 2 weeks). It is important to recall that at this time the fetal testes, adrenal glands, kidney and associated tissues are located very close together on each side of the body, such that mutual hormonal influences are largely of a paracrine nature within one side (34). The contralateral organs are further away. For such paracrine systems, only very low concentrations of a hormone are sufficient to activate G-protein-coupled receptors such as RXFP2, well below the 10% effective concentration (EC^10^) for the receptor (<10^-10^M) and, being locally produced, there may not yet be sufficient hormone to be detected in the fetal bloodstream, or in amniotic fluid. Thus, we can assume that the fetal INSL3/RXFP2 system will be activated before INSL3 becomes measurable in fetal blood or in amniotic fluid. **Figure 2** indicates INSL3 mRNA determined by RNA microarray analysis in samples of human fetal gonads collected during the first trimester (35) and indicates an up-regulation in testes only, and not in ovaries, at weeks 7-8 pc. The upregulation of INSL3 follows precisely (i.e. actually on the same day during fetal development) the concomitant upregulation of the genes necessary for androgen production in the testes (34). If constitutively expressed, we can assume that INSL3 peptide will be produced almost immediately following gene transcription. This probably increases after that, since other studies report highest fetal testis INSL3 mRNA at around gestation weeks 17-18 (36), though it is expressed earlier.

There is limited data available for the human in regard to the INSL3 concentration in fetal blood. Harrison and colleagues reported 0.44-2.04 ng/ml from umbilical cord venous blood in gestation weeks 15-20 (37). Lower concentrations of INSL3 are recorded in cord blood at term of pregnancy [control subjects: France, 0.27 ± 0.18 ng/ml (38); Japan, 0.28 (0.25-0.32 IQR) ng/ml (39); Denmark, 0.13 (<0.05-0.34 range) ng/ml (40); Finland, 0.14 (0.06-0.39 range) ng/ml (40)], with the small differences probably attributable to the different assays being used to measure INSL3.

**Figure 3:**
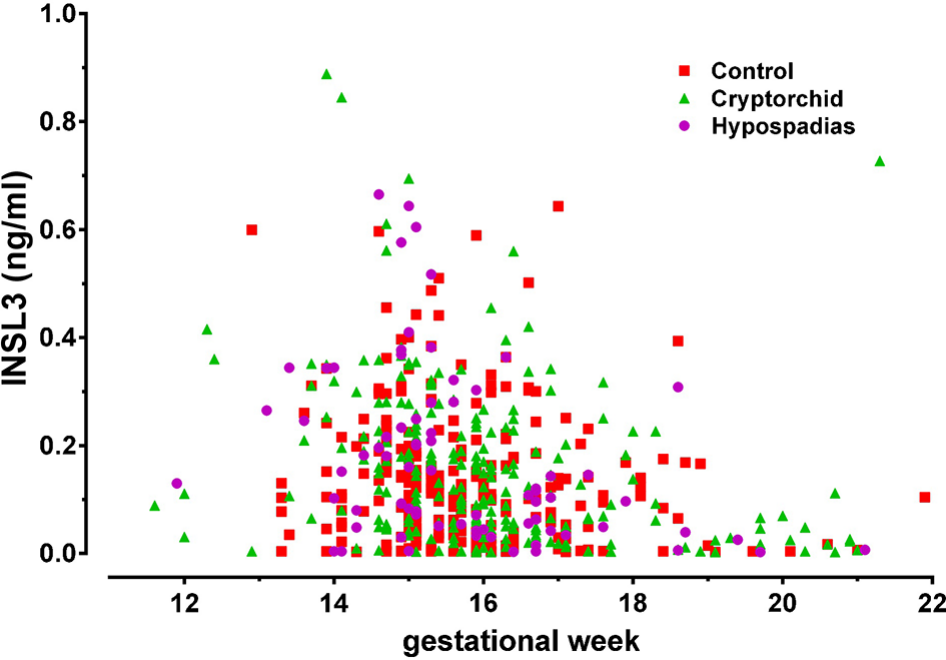
INSL3 concentration measured by specific time-resolved fluorescent immunoassay from human amniotic fluid samples collected at routine amniocentesis at the times indicated. The pregnancies were identified postnatally as normal (control), cryptorchid, or hypospadias [reproduced from (42)].

INSL3 can also be detected in human amniotic fluid that has been routinely collected at amniocentesis for prenatal genetic diagnosis (41, 42). It is measurable only for male fetuses (41) and in the earliest sample available already at gestation week 11, though reaches a maximum at gestation weeks 12-16 (**Figure 3**). It appears to decline to undetectable levels by about week 20. Whilst this might reflect a reduced Leydig cell production at that time, it is also probable that skin keratinization at about gestation week 20 leads to a limitation of peptides and proteins being exuded from fetal blood *via* the fetal skin. After this time, the constitution of amniotic fluid more likely reflects the products of the fetal lungs and kidneys, as well as of the amniotic membranes themselves.

INSL3 has also been measured in fetal fluids from rats (43), pigs (44), and cows (45), where the timing of INSL3 expression largely mirrors the dynamics of testicular descent which, for example, occurs shortly after parturition in rodents, unlike in humans or cows, where it occurs earlier during the second trimester (45). These studies were also able to show that INSL3 from the testes of male fetuses was able to cross the placental barrier either to the maternal circulation [cows (45)] or to neighboring female fetuses [pigs (44)]. The mechanism of such transfer is unknown, but in analogy to insulin would suggest some kind of mediating molecular transport system.

## The Role of INSL3 in Testicular Descent

Inactivation of the genes encoding INSL3 or its receptor, RXFP2, in mice leads to the same essential phenotype, namely bilateral cryptorchidism with a failure of the first transabdominal phase of testicular descent (11, 12, 46). Similar bilateral cryptorchidism was also achieved in the male offspring of pregnant rats treated with a specific RXFP2 competitive antagonist (47). Genetic evidence in humans is more problematic. Since both genes are autosomally recessive, mutations in single alleles rarely lead to cryptorchidism. Several population studies, however, have indicated an association between heterozygous mutation in either INSL3 or RXFP2 and the incidence of cryptorchidism (usually unilateral) (48, 49). Because cryptorchidism, unless corrected, inevitably leads to male infertility such deleterious mutations are historically at a low frequency.

Testicular descent is a highly dynamic process. Prior to gonadal sex determination, the indeterminate gonad lies adjacent to the fetal kidney and adrenal complex (mesonephros) apposing the dorsal wall of the coelomic cavity. It is attached dorsally to the body wall by the cranial suspensory ligament (CSL) and ventrally to the body wall in the inguinal region by the gubernacular ligament. Initially, both ligaments are relatively short and undeveloped. In the following days, the fetus grows with the kidney moving in an antero-dorsal direction relative to the inguinal region. In the male fetus, testosterone produced by the differentiating Leydig cells causes the CSL to involute, becoming longer and thinner. In contrast, INSL3 produced by the same cells causes the mesenchymal core of the gubernacular ligament to shorten and expand laterally to form the gubernacular bulb, which now retains the fetal testis in the inguinal region as the kidney and adrenal grow away in an antero-dorsal direction (18, 50). Thus, this first phase of testicular descent does not involve any actual ventral movement of the fetal gonads, but merely a retention of the gonad in the inguinal region. Subsequently, in the second phase of descent, the gubernacular bulb everts through an inguinal weakening of the body wall, creating an inguinal canal and causing the fetal testes to relocate into the scrotum (18). This second phase may also require some INSL3 in addition to androgens (16). In the female fetus, the CSL fails to involute, retaining the ovary near the kidney; there is also no INSL3 produced and hence no gubernacular thickening and no gonadal ‘descent’. In the male *tfm* (testicular feminization) mouse, there are no functioning androgen receptors, and hence the CSL remains thick and fails to involute, but the INSL3 still induces gubernacular development, with the result that in this mouse the fetal testis is held by the two ligaments within the abdomen in an intermediate location as if on a ‘taut bowstring’. In the INSL3 knockout mouse, the opposite occurs, with neither ligament developed and the fetal testes appearing to be loosely swimming within the peritoneal cavity (11). In female fetal mice, which have been genetically engineered to produce INSL3, it appears that besides causing a slight dislocation of the ovaries, INSL3 also induces abdominal hernia (51), suggesting that the INSL3/RXFP2 system may additionally be involved in aspects of the second inguino-scrotal phase of testis descent in the males. It is to be noted though that RXFP2 expression in female mice is likely to be much reduced because of less androgen production (see below).

Whether the INSL3/RXFP2 hormonal system is involved in later stages of testicular descent is unclear since disruption of the first phase inevitably leads to a disruption of the relative dynamics of testicular descent as a whole, including of subsequent phases. This is a very active stage of fetal development. Anatomical examination of unilateral and bilateral cryptorchidism often indicates that other tissues or ligaments have become interposed possibly because of altered relative timing of their growth trajectories. Certainly, it is understood that androgens as well as neural input from the CGRP-expressing genito-femoral nerve are principally involved in the second inguinal-scrotal phase of testis descent, at least in rodents (18), though *in vitro* studies suggest that both INSL3 as well as AMH may also have a role (52). Cryptorchidism (unilateral or bilateral) is very common in the male population and hints at more than the involvement of one or two simplistic regulatory disorders; it has recently been suggested that it is indeed a neuro-humoral multifactorial syndrome involving a dynamic network of a range of diverse factors (53). However, it should be noted that altogether much less is known about the situation in humans compared to experimental animals. Because the early left and right organ complexes are discrete from one another and each appears to be regulated separately by local paracrine factors during testicular descent, this might explain the preponderance of unilateral cryptorchidism, whereby only one such complex is dynamically disrupted.

Whether INSL3 is also involved in other fetal processes other than testis descent is not known, nor whether RXFP2 is also expressed in other fetal tissues than the gubernaculum. An exception here is provided by horn buds in male ruminants, where RXFP2 is expressed within the horn bud and mutations in the RXFP2 gene are associated with polledness in sheep and cattle (54). More research is needed here; it seems likely that the INSL3/RXFP2 system may well be involved in areas of fetal physiology with significant sex-specific aspects. This might prove relevant, for example, in cases of twinning with male and female fetuses sharing the same uterine environment (see above), although studies of such effects to date are still ambiguous (55).

## The Regulation of INSL3 and RXFP2

As mentioned earlier, the upstream promoter region of the INSL3 gene in most species evaluated appears to be relatively short, encompassing maximally 1000bp. Specifically, it includes three discrete responsive elements for the transcription factor steroidogenic factor 1 (SF1) (32, 56), and *in vitro*, transfected promoter-reporter constructs achieve maximal activity simply by co-transfecting the unmodified transcription factor (27, 32). All three SF1-responsive elements (SFREs) appear to be functional (32, 56). This could be enough to explain the up-regulation of INSL3 in fetal Leydig cells *in vivo* which appears to occur immediately following the expression of SF1 (35) in the same cells, and in these as well as in adult Leydig cells appears to occur constitutively. For example, there is full INSL3 expression in the fetal Leydig cell population of the *hpg* (hypogonadal) mouse in which the HPG axis is disrupted because of an absence of GnRH and hence LH (6). However, whether all three SFREs are normally occupied by SF1 is unclear, as is also whether or not other related transcription factors may also compete for binding to the gene promoter. An SFRE can also bind and respond to the closely related transcription factor Nur77, which at least in adult Leydig cells appears to be principally involved in INSL3 up-regulation (57). It is also recognized that the inhibitory transcription factor COUP-TF may also bind to an SFRE and compete with SF1 to control the up- or down-regulation of a gene (58); and it is now established that COUP-TF may play an important role in the differentiation and development of the fetal testis (59) as well as in regulating the INSL3 gene (60). However, it has also been shown that several nuclear steroid receptors may also influence gene expression using SFREs, probably in a non-classical manner which does not involve direct interaction with the responsive element in the DNA itself (61). Both rodent and bovine INSL3 promoter-reporter constructs can be stimulated *in vitro* by activated estrogen and androgen receptors (56, 62), though whether *via* classical or non-classical mechanisms is not clear. If this is relevant and important also for fetal INSL3 expression is not known. It should also not be forgotten that INSL3 expression is extremely cell-type specific. In the fetus, there is expression only in a subset of fetal Leydig cells (63), and not in any other testicular cell although, for example, fetal Sertoli cells also express SF1. Nor is there any expression in steroidogenic adrenal cells even though these share a similar mesonephric mesenchymal origin as fetal Leydig cells. There are evidently specific elements within the INSL3 gene which determine high cell-type specificity.

Very little is known about the regulation of RXFP2 expression. Indirect evidence from mice in which the LH receptor gene has been disrupted implies that RXFP2 expression requires activated androgen receptors to induce the appropriate level of the RXFP2 receptor (64). What is also evident is that in the male fetus (at least in rodents) its expression is also specifically restricted to certain cell types only (17), such as those of the mesenchymal core of the gubernaculum.

## INSL3 as a Monitor of Environmental Endocrine Disruption

**Figure 4:**
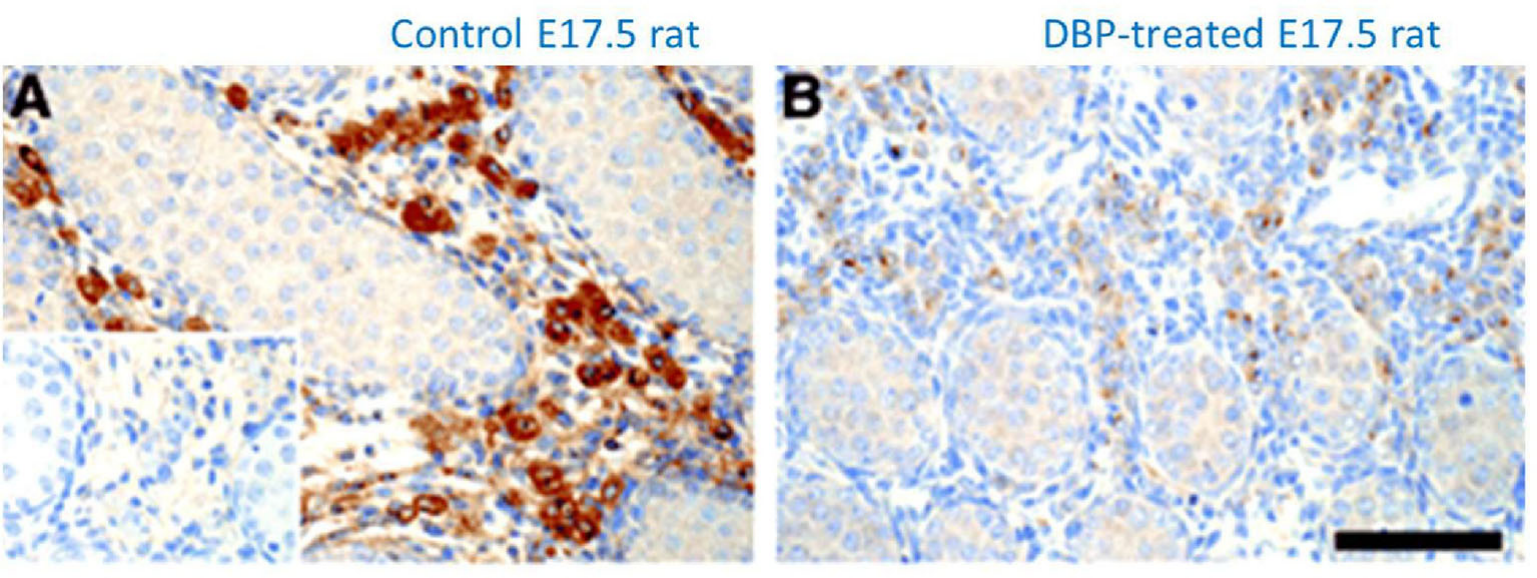
Immunohistochemical staining for INSL3 (brown color) in the fetal testes on gestational day E17.5 of male rats which had been maternally exposed to dibutyl phthalate **(B**; DBP**)** or vehicle **(A)** during the important window for male development (E12-E16). Control section using pre-immune serum is indicated in the bottom left of panel **(A)** [reproduced from (65)].

INSL3 represents a major secretory product uniquely from the male fetus and not from either a female fetus or the mother, at a time in the human during the transition from first to second trimester, when most organ systems are developing and differentiating most rapidly. It thus offers to be an excellent biochemical biomarker for such early organogenesis, particularly as this is a period in pregnancy which is otherwise difficult to monitor (43). Numerous studies in pregnant rats have shown that maternal exposure to a variety of environmental endocrine disrupting chemicals (EDCs) leads to a reduction in INSL3 mRNA or protein expression by the fetal Leydig cells (**Figure 4**) with disruption of the male reproductive phenotype, including cryptorchidism, hypospadias, and reduced anogenital distance (65–67). These chemicals include phthalate esters (**Figure 4**), used as plasticizers or previously in cosmetics, as well as xenoestrogens, such as diethylstilbestrol or bisphenol A (67, 68). The chief mode of action of these substances appears to be to disrupt fetal Leydig cell differentiation leading to a reduction in testosterone production and/or INSL3. Importantly, even after only a brief maternal exposure, these substances also appear to affect Leydig stem cells which reside within the testes even into adulthood, and hence may also influence puberty and adult Leydig cell function (67, 69).

For the human, studies have compared second trimester INSL3 concentration in amniotic fluid with the levels of phthalates and PFOS. Investigations in a large Danish biobank showed at the population level that INSL3, and hence Leydig cell function, was significantly reduced in proportion to the EDC load (42, 70, 71). Similarly, other studies of INSL3 in cord blood at term of pregnancy also indicated a significant negative relationship between phthalate or bisphenol A exposure and INSL3 concentration (39, 72). Similar studies have also indicated that INSL3 concentration in term cord blood is significantly different between control male infants and those exhibiting cryptorchidism (38), implying that at least at a population level these three factors (EDC load, INSL3, cryptorchidism) are mechanistically linked, presumably *via* the common element of fetal Leydig cell function. It is to be noted though that INSL3 in second trimester amniotic fluid does not appear to differ significantly between normal male infants and those born with cryptorchidism or hypospadias (**Figure 3**) (42).

Explanted human fetal testis fragments have also been assessed either using *in vitro* culture (73) or following xenotransplantation to immune-compromised mice (74). Again, EDC exposure suggests a disruption of fetal Leydig cell differentiation and/or functionality. The most commonly used EDC in these experiments is represented by the phthalate esters which are widespread especially in the environment through their inclusion in numerous plastics, coatings, and cosmetics. More recently, however, the focus has shifted to show that also common analgesics, such as acetoaminophen (paracetamol) or ibuprofen, which are widely taken to alleviate pain during pregnancy, appear to have a similar impact on the development of fetal Leydig cells and the expression of INSL3 (74, 75).

What is important in these investigations and is being reinforced through several studies in rats (67, 76–78), is that the impact of these EDCs is less an acute one acting, for example, *via* modulation of steroid receptors, but rather an effect which is altering the differentiation dynamics of Leydig cell precursors. Both in the fetus and during puberty, and possibly also in later life, Leydig cells are developing *via* the two processes of proliferation and differentiation held in fine balance with one another. Perturbation of either of these processes will lead to an altered final Leydig cell functional capacity, and hence to an altered capacity to produce testosterone and other hormones essential to maintain health in later life. INSL3 is an accurate measure of this Leydig cell functional capacity (5) and can monitor the impacts of EDCs and other exogenous factors, besides having endocrine functions in its own right, for example, to improve bone quality (79).

## Conclusion

INSL3 is a major secreted hormone produced by the Leydig cells of the fetal testis, shortly after its differentiation from the undetermined gonad. This occurs immediately following gonadal sex determination and the expression of the transcription factor SF1. Its main function in the male fetus is to induce thickening of the gubernacular ligament anchoring the testes in the inguinal region, thereby promoting the first transabdominal phase of testicular descent. As a major male fetal hormone in the first and second trimesters of human pregnancy, it likely also has other roles about which we still have little information. Importantly, it can also act as a biomarker for fetal physiology in this relatively obscure phase of pregnancy, responding to maternal exposures such as to EDCs or to analgesic pharmaceuticals.

## Author Contributions

All authors have contributed equally to the conception, writing, and editing of the manuscript.

## Conflict of Interest

The authors declare that the research was conducted in the absence of any commercial or financial relationships that could be construed as a potential conflict of interest.

## Publisher’s Note

All claims expressed in this article are solely those of the authors and do not necessarily represent those of their affiliated organizations, or those of the publisher, the editors and the reviewers. Any product that may be evaluated in this article, or claim that may be made by its manufacturer, is not guaranteed or endorsed by the publisher.
